# The Roles of Plant-Growth-Promoting Rhizobacteria (PGPR)-Based Biostimulants for Agricultural Production Systems

**DOI:** 10.3390/plants13050613

**Published:** 2024-02-23

**Authors:** Wenli Sun, Mohamad Hesam Shahrajabian, Ali Soleymani

**Affiliations:** 1 National Key Laboratory of Agricultural Microbiology, Biotechnology Research Institute, Chinese Academy of Agricultural Sciences, Beijing 100081, China; hesamshahrajabian@gmail.com; 2 Department of Agronomy and Plant Breeding, Isfahan (Khorasgan) Branch, Islamic Azad University, Isfahan 81551-39998, Iran; a.soleymani444@gmail.com; 3 Plant Improvement and Seed Production Research Center, Isfahan (Khorasgan) Branch, Islamic Azad University, Isfahan 81551-39998, Iran

**Keywords:** *Acinetobacter*, *Arthrobacter*, biostimulants, *Enterobacter*, *Ochrobactrum*, *Pseudomonas*, *Streptomyces*

## Abstract

The application of biostimulants has been proven to be an advantageous tool and an appropriate form of management towards the effective use of natural resources, food security, and the beneficial effects on plant growth and yield. Plant-growth-promoting rhizobacteria (PGPR) are microbes connected with plant roots that can increase plant growth by different methods such as producing plant hormones and molecules to improve plant growth or providing increased mineral nutrition. They can colonize all ecological niches of roots to all stages of crop development, and they can affect plant growth and development directly by modulating plant hormone levels and enhancing nutrient acquisition such as of potassium, phosphorus, nitrogen, and essential minerals, or indirectly via reducing the inhibitory impacts of different pathogens in the forms of biocontrol parameters. Many plant-associated species such as *Pseudomonas*, *Acinetobacter*, *Streptomyces*, *Serratia*, *Arthrobacter*, and *Rhodococcus* can increase plant growth by improving plant disease resistance, synthesizing growth-stimulating plant hormones, and suppressing pathogenic microorganisms. The application of biostimulants is both an environmentally friendly practice and a promising method that can enhance the sustainability of horticultural and agricultural production systems as well as promote the quantity and quality of foods. They can also reduce the global dependence on hazardous agricultural chemicals. Science Direct, Google Scholar, Springer Link, CAB Direct, Scopus, Springer Link, Taylor and Francis, Web of Science, and Wiley Online Library were checked, and the search was conducted on all manuscript sections in accordance with the terms *Acinetobacter*, *Arthrobacter*, *Enterobacter*, *Ochrobactrum*, *Pseudomonas*, *Rhodococcus*, *Serratia*, *Streptomyces*, Biostimulants, Plant growth promoting rhizobactera, and *Stenotrophomonas*. The aim of this manuscript is to survey the effects of plant-growth-promoting rhizobacteria by presenting case studies and successful paradigms in various agricultural and horticultural crops.

## 1. Introduction

Biostimulants can be used to complement the application of chemical inputs, including the utilization of beneficial rhizosphere microbiome like advantageous fungi and plant-growth-promoting rhizobacteria [1,2,3]. The major biostimulants effects on crops include improving the visual quality of final products, stimulating the immune systems of plants, inducing the biosynthesis of plant defensive biomolecules, removing heavy metals from contaminated soil, improving crop performance, reducing leaching, improving root development, improving seed germination, inducing tolerance to abiotic and biotic stressors, accelerating crop establishment, and promoting nutrient uptake and nutrient use efficiency [4,5]. Biostimulants are components that increase plant growth but do not qualify as essential plant nutrients, but biofertilizers are live microbes whose primary impact is to increase plant growth. Moreover, as biofertilizers are live microbes whose primary influence is to increase crop growth, biopesticides are live organisms whose primary effect is to directly control and manage crop diseases and pests. It is important to consider the point that the main difference between biostimulants and biofertilizers is that biofertilizers contain many nutrients but biostimulants do not have the plant nutrients. *Ochrobactrum* species are Gram-negative, non-enteric, non-fermenting bacteria that are closely associated with the genus *Brucella*, which are found in wide range of environments including in animals, plants, soil, aircraft, and water [6,7]. Sipahutar and Vangnai [8] observed that *Ochrobactrum* sp. MC22 can improve the yield of soybean and mung bean with significant function for rhizoremediation in a crop area with triclocarban contamination. *Acinetobacter* is a Gram-negative bacterium found in nature, especially in the rhizosphere of many plants, playing an important function as a plant-growth-promoting bacterium and being known to produce gibberellin, siderophore, IAA, biosurfactants/bioemulsifiers, and antibiotics, as well as solubilize zinc, potassium, and phosphate. Bacteria of the *Enterobacter* species belong to the ESKAPE (*Enterobacter* spp., *Pseudomonas aeruginosa*, *Acinetobacter baumannii*, *Klebsiella pneumoniae*, *Staphylococcus aureus*, and *Enterococcus faecium*) group of pathogens [9,10,11]. It has been reported that microbial biostimulants can synthesize IAA, which can promote root branching and plant growth of the plants under both abiotic and biotic stresses [4,5]. Microbial biostimulants can alleviate salt stress as they are associated with a high level of IAA and can improve them in the re-establishing of favorable water potential gradients under water shortage conditions as well as increasing film hydration around the roots [1,2,3,4,5]. Both PGPR and plants have ACC-deaminase, which has the ability to reduce the concentration of ethylene in the root zone and roots, with PGPR-derived ACC-deaminases being able to decrease ethylene-induced inhibition by reducing root zone ethylene [12,13,14]. *Enterobacter* species notably increased below- and aboveground responses in rice plants [12]. *Arthrobacter* species are obligate aerobes and Gram-positive chemoorganotrophs that are often found among soil bacteria [13], which is a major aerobic bacterium under the class of *Actinobacteria* and the family *Micrococcaceae* [14]. The most well-known and important in situ bioremediation of them is Cr(VI) reduction abilities [15]. The biomass of *Arthrobacter protophormiae* was used to detach Cd(II) from an aqueous solution [16]. *Arthrobacter echigonensis* MN1405 helped *Phytolacca acinosa* Roxb. in obtaining high remediation effectiveness of Mn removal and accumulation in the Mn contamination area [17]. *Pseudomonas* are known as plant-associated and soil-dwelling species because of their biological activity of controlling plant diseases, both indirectly through inducing plant defense resistance responses or directly via producing antagonistic metabolites, especially by their large class of secondary metabolites, which are known as cyclic lipopeptides [18]. Different species of the *Pseudomonas* genus are well known as exhibiting plant growth promotion traits, such as indole acetic acid (IAA) biosynthesis, phosphate solubilization, and stress alleviation enzyme production, which are important characteristics for the development of effectual plant biostimulants [19]. *Rhodococcus* spp. are a group of non-model Gram-positive bacteria that have various catabolic activities with high adaptive capabilities, making them unique because of different applications in lignocellulosic biomass conversion, environmental bioremediation, and whole-cell biocatalysis [20]. This genus includes a small number of opportunistic pathogens and species, making them appropriate from the point of view of safety [21].

*Serratia* is a Gram-negative, rod-shaped bacterium that is an important ubiquitous member of the *Enterobacteriaceae* family [22]. *Serratia proteamaculans* suppressed *Rhizoctonia solani* in vivo and in vitro, increased plant growth parameters, and stimulated tomato defense machinery [23]. Kang et al. [24] reported that *Serratia nematodiphila* PEJ1011 can regulate the endogenous ABA levels in pepper plants while reducing the endogenous salicylic acid and jasmonic acid contents. *Streptomyces* spp. is a filamentous and Gram-positive prokaryote, being the main clade of the phylum *Actinobacteria*, belonging to the family of *Streptomycetaceae* [25], which are ubiquitous in marine sediments and soils; moreover, they are usually found inside plant roots and in the rhizosphere [26]. It is the most well-known genus of *Actinobacteria*, being a Gram-positive mycelial sporulating bacteria with a great ability to be resident in dry and saline soils or live within spontaneous plants of drylands, with the ability of producing IAA and siderophores, as well as solubilizing mineral phosphates [27]. It can be dispersed by arthropods, insects, and other microbes, and it contains unique metabolites that can influence insect behavior and bacterial growth [28]. The complexity of soil environments and the interactions of *Streptomyces* with other organisms is the main reason for the production of secondary metabolites [29]. They have shown great potential for protection against fungal disease, plant growth promotion, and colonization ability in cereals [30]. Kaari et al. [30] concluded that *Streptomyces* sp. UT4A49 can be considered as a promising biocontrol factor for tomato bacterial disease control, that of *Ralstonia solanacearum*. Elango et al. [31] reported that *Streptomyces lydicus* and *Streptomyces griseus,* together with *Trichoderma harzianum* and *Bacillus subtilis,* are recommended for the control of red root rot disease of tea plants. Some of the most important *Streptomyces* with biocontrol activities are *Streptomyces griseus*, *Streptomyces kasugaensis*, *Streptomyces* J-1, *Streptomyces* sp., *Streptomyces sanglieri*, *Streptomyces griseorubens* E44G, *Streptomyces rochei* ACTA1551, and *Streptomyces felleus* YJ1, and notable *Streptomyces* with plant-growth-promoting activities are *Streptomyces anulatus* S37, *Streptomyces* sp., *Streptomyces matansis* BG5, *Streptomyces* sp. RSF17, *Streptomyces vinaceus* CRF2, *Streptomyces pulcher* CRF17, *Streptomyces* PRIO41, *Streptomyces mutabilis*, and *Streptomyces fumangs* gn-2 [32,33]. *Stenotrophomonas* is one of the most important aerobic plant-growth-promoting bacterium with around 18 well-characterized species, having shown a high capability in phosphate solubilization, nitrogen fixation, siderophore production, the production of plant growth regulators, and antagonism against pathogenic microorganisms [34]. Its species also have important functions in the bioremediation process by helping in biofortification and degrading xenobiotic compounds, as well as having significant roles in the improvement of crop plant health [35]. The action mechanisms of microbial biostimulants in plants are not well understood yet; however, PGPR can upregulate the expression of genes related to cell growth and cellulose biosynthesis; promote shoot length; increase water use efficiency, photosynthesis, and water retention in drought conditions; increase gas exchange; promote plant biomass; decrease the levels of lipid peroxidation; increase organic acid, protein, soluble sugar, and biomass production; improve chlorophyll, ABA levels, and compatible solutes; and enhance the activity of antioxidant enzymes as well as reducing membrane permeability in salt stress conditions [3,4,5,6,7,8,9,10]. The goal of this review article is to survey the impacts of plant-growth-promoting rhizobacteria by presenting case studies and successful paradigms in different agricultural and horticultural crops. The title of this article was selected as PGPR has an important role in plant growth through direct action mechanisms, and understanding the roles and impacts of different types of PGPR is important in terms of achieving more sustainable agricultural goals. In this article, we also tried to study the major mechanisms of action of different types of biostimulant products with an emphasis on the application and integration of microbial-based biostimulant products in horticultural and agricultural crop production. This research examines the scientific literature on biostimulants from 1991 to December 2023 by conducting a bibliometric analysis of the literature published on the Web of Science database, including more than one thousand articles. The information provided was obtained from randomized control experiments, review articles, and analytical observations and studies that have been gathered from various literature sources such as PubMed, Science Direct, Scopus, and Google Scholar. The keywords used were the Latin and common names of different agricultural and horticultural species, as well as microbial biostimulants, such as “*Ochrobactrum*”, “*Acinetobacter*”, “*Arthrobacter*”, “*Enterobacter*”, “*Pseudomonas*”, “*Rhodococcus*”, “*Serratia*”, “*Streptomyces*”, “Biostimulants”, “Plant growth promoting rhizobactera”, and “*Stenotrophomonas*”.

## 2. *Ochrobactrum* spp.

*Ochrobactrum* spp. belongs to the *Brucellaceae* family and is a class-alpha-proteobacteria [36], commonly identified in the soil within the roots of a plant [37]. *Ochrobactrum* spp., together with the closely related *Brucella*, *Agrobacterium*, and *Rhizobium* genera, belong to the class of *Alphaproteobacteria* [36,37], which are also of interest as plant beneficial bacteria, as some of the strains are able to nodulate roots to fix nitrogen, underlining their close association with the host plants. They can also improve the germination percentage, as well as increase the protease activities, amylase, and relative root elongation, and they can be considered as important in bioremediation as well as an important alternative for plant growth promotion. *Ochrobactrum* is usually found in entomopathogenic nematode symbiotic systems, and it has been reported that the *Ochrobactrum intermedium* strain produces glyco lipopeptide biosurfactant [38]. Ochrosin, which is a multi-functional bio-surfactant produced by *Ochrobactrum* sp. BS-206, shows appropriate anti-adhesive, antimicrobial, antifeedant, and insecticidal activities [39]. *Ochrobactrum* sp. MPV1 is an appropriate candidate for the bioconversion of toxic oxyanions, including tellurite and selenite, to their respective elemental forms, producing intracellular Se and TeNPs possibly accessible in industrial and biomedical applications [40]. *Ochrobactrum anthropi* DE2010 indicated high tolerance and a high removal ability value for Cr(III), as well as being effectual in immobilizing Cr(III) [41]. *Ochrobactrum* JAS2 isolated from paddy rhizosphere soil indicated meaningful advantages of the production of hydrogen cyanide, ammonia, and IAA with significant plant-growth-promoting capabilities [42]. *Ochrobactrum lupini* KUDC1013 consisted of systemic resistance against spots caused by *Xanthomonas axonopodis* pv. *vesicatoria* in pepper, as well as resistance against leaf sport rot caused by *Pectobacterium carotovorum* subsp. *carotovorum* in tobacco [43]. The influence of different kinds of *Ochrobactrum* on experimental plants are shown in Table 1.

## 3. *Acinetobacter* spp.

*Acinetobacter* spp. is a Gram-negative coccobacilli that is aerobic and non-motile, with no glucose-fermentation capability, being found in different environments [59,60,61,62,63]. One of the main reasons for the study of the genus *Acinetobacter* is because of its role in antimicrobial resistance and serious infections [62]. It can fix nitrogen, solubilize minerals, produce siderophores, and even be used as plant epiphytes or endophytes, allowing it to assist hosts in detaching pollutants and tolerating environmental stresses [63]. It is short, plump, and typically 1.0–1.5 μm by 1.5–2.5 μm in size during the rapid phase of its growth [64,65]. Shah et al. [66] observed that zinc sulfide nanoparticles, *Bacillus velezensis*, and *Acinetobacter pittii* have great potential against *Rhizoctonia solani* in tomato to suppress root rot infection and improve yield and growth. They have also found that this combination can increase tomato plant nutrition such as in terms of potassium, calcium, silicon, and magnesium, as well as improve redox quenching status by increasing the activity of antioxidant defense enzymes [66]. Wang et al. [67] also discovered that *Acinetobacter oleivorans* S4 is important for plant growth and valuable in assisting in phytoremediation. Ke et al. [68] reported that the *Acinetobacter indicus* strain ZJB20129 isolated from an urban sewage treatment plant showed a heterotrophic nitrification-aerobic denitrification ability. *Acinetobacter* sp. TX5 has a valuable ability in terms of nitrite removal with a capability of suppressing N_2_O accumulation, and it has been reported that *Acinetobacter* sp. XS21 effectively removes arsenite from soluble-exchangeable fraction [69], with *Acinetobacter calcoaceticus* strains from canola and soybean having been reported to improve plant growth [70]. The improved seedling growth parameters of the treated crop seeds of *Vigna unguiculata*, *Vigna radiata*, *Dolichos lablab*, and *Abelmoschus esculentus* showed the wonderful potential of *Acinetobacter* sp. RSC7 to be used in a bio-fertilizer formulation in a sustainable production system [71]. The strain *Acinetobacter calcoaceticus* DD161 has high inhibitory activity against the *Phytophthora sojae* 01 in soybean [72]. *Acinetobacter* sp. strain Xa6 can be used as a biological control against *Ralstonia solanacearum* as well as increase the final yield of tomato [73]. *Acinetobacter* sp. strain SG-5 is an important candidate as a metal remediation plant, particularly in terms of Cd in edible plant parts and as a plant growth promoter [74].

## 4. *Arthrobacter* spp.

The genus *Arthrobacter* can effectually utilize inorganic and organic compounds as a metabolism substrate, acting as a tool for bioremediation in agriculture [75,76,77,78]. Certain *Arthrobacter* species, mainly soil-dwelling rhizobacteria, have been considered as plant growth promoters because of their different growth-promoting activities, such as potassium and phosphate solubilization, nitrogen fixation, and indole acetic acid synthesis [79,80]. Zhao et al. [81] reported that *Arthrobacter* sp. ZCY-2 has a circular chromosome and five circular plasmids encoding for the procedure of salt adaptation and pollutant degradation, proving its unique saline tolerance characteristic. Zhao et al. [82] reported that Ap920-WI from *Arthrobacter* sp. H5 has shown high anti-fungal activity and that it inhibited the infestation of *Sclerotinia sclerotiorum* on rape leaves, with it potentially offering a novel solution for controlling plant diseases. The effects of *Arthrobacter* on various plants are presented in Table 2.

## 5. *Enterobacter* spp.

The genus *Enterobacter* is a member of the family *Enterobacteriaceae* of the class *Gammaproteobacteria* [97], and the *Enterobacteriaceae* family includes different bacteria such as *Enterobacter* spp., *Klebsiella* spp., and *Escherichia coli* [98,99,100]. Ngigi et al. [101] found that the existence of bacterial strains such as *Enterobacter cloacae* in different sugarcane-cultivated fields can be used to increase the degradation of atrazine in contaminated soil, where atrazine is known to be recalcitrant. *Enterobacter roggenkampii* ED5 has notable application potency in sugarcane production as it can boost plant growth, as well as being able to enhance photosynthetic leaf gas exchange capacity and agronomic trains in sugarcane [102]. Mayak et al. [103] also reported that the plant-growth-promoting bacterium *Enterobacter cloacae* CAL3 has a positive influence on tomato seedlings. Ullah et al. [104] reported that the Zn-solubilizing endophyte *Enterobacter* sp. MN17 with Zn utilization via seed coating can increase the profitability, productivity, bioavailable Zn, and grain quality of *Kabuli* chickpea. Utkhede and Smith [105] also noted that *Enterobacter agglomerans* is appropriate for the control of crown and root rot of apple trees. Zakria et al. [106] also observed the positive effects of *Enterobacter* sp. strain 35-1 and *Enterobacter* sp. SE-5 isolated from sugarcane on both rice (*Oryza sativa*) and wild rice (*Oryza officinalis*). The beneficial impacts of *Enterobacter* species on potassium, nitrogen contents, leaf area, stem girth, and seedling height proved its important role as a bio-inoculant for maize seedling growth and health [107].

*Enterobacter hormaechei* subsp. *steigerwaltii* EB8D was able to improve the root length, stem length, and dry weight of tomato plants, as well as indicating a significant strong protective capability against *Fusarium oxysporum* f.sp. *radicis lycopersici* [108]. Zakria et al. [109] reported that the positive impacts of *Enterobacter* sp. strain 35-1 on *Brassica oleracea*, while Mowafy et al. [110] reported that the significant influence of *Enterobacter* on final yield and root nodulation of maize and soybean, respectively. *Enterobacter sichuanensis* AJI 2411 showed growth-promotion properties for plants, being isolated from the rhizosphere of soybean [111]. Ji et al. [112] reported that *Enterobacter cloacae* HG-1 could promote the growth of wheat under salt stress and increase salt stress tolerance. Garcia-Gonzalez et al. [113] found that *Enterobacter cloacae* can be considered as an emerging plant-pathogenic bacterium that positively affects chili pepper seedlings, and Abedinzadeh et al. [114] found that *Enterobacter cloacae* subsp. *cloacae* ATCC13047^T^ is a causal agent of decline and offshoot rot of the date palm in North Africa and Southwest Asia. Taha et al. [115] found that *Rhizobium laguerreae* co-inoculated with native *Bacillus* sp. and *Enterobacter aerogenes* improved areal biomass and shoot dry weight and mitigated the drought stress impact on lentil. *Enterobacter* sp. EG16 can decrease physiological stress and promote pak choi (*Brassica rapa* spp. *Chinensis*) growth and nutritional quality [116]. *Enterobacter cloacae* CTWI-06 can mitigate the negative effects of Cr(VI) in rice plants as it was both resistant to elevated Cr(VI) concentration and a wide array of other metals [117]. *Enterobacter cloacae* ZA14 can boost the growth of tomato by mitigating the phytotoxicity of dyes and heavy metals from textile wastewater, improve the metal tolerance index, and increase the production of total thiols [118]. *Enterobacter asburiae* NC16 decreased transpiration rates and the expression of some iron (Fe)-uptake-related genes such as *ZmNAS2*, *ZmZIP*, *ZmYS1*, and *ZmFer* in corn plant, having an important function in the mitigation of Cd toxicity, partially by hampering the Fe-uptake-associated pathways [119]. *Enterobacter* sp. TY-1 is an important Cd-resistant strain with notable plant growth promotion traits that can induce to improvement in dry weight, fresh weight, plant height, and Cd accumulation of ryegrass [120], whereas *Enterobacter ludwigii* was found to improve the nodulation, yield, and quality of alfalfa under saline-alkali environments [121]. The endophytic bacterium *Enterobacter cloacae* (MG001451) isolated from *Ocimum sanctum* showed high potency in terms of antimicrobial activities [122]. Rani et al. [123] reported that the application of *Enterobacter ludwigii*-PS10 can significantly increase Zn content and plant biomass of tomato plants, as well as increasing bacterial cell viability and plant growth.

*Enterobacter roggenkampii* Kh2 is the causal agent of postharvest sugar beet soft rot disease [124], and *Enterobacter* sp. FM-1 (FM-1) had a meaningful impact on the rhizosphere soil N and C cycles on *Bidens pilosa* L. in heavy-metal -contaminated soil [125]. In another experiment, *Klebsiella* sp. Z2 and *Enterobacter* sp. Z1 improved soybean nodule production with rhizobia; increased the taurine, phytohormone, and flavonoid contents of soybean; and secreted phytohormone to activate flavonoid synthesis gene expression [126]. *Enterobacter* sp. alleviated salinity stress in *Cajanus cajan* and plant growth enhanced by the application of 1-aminocyclopropane-1-carboxylic acid (ACC)-deaminase-positive bacteria [127]. In another trial, *Enterobacter* sp. E1 effectively boosted As accumulation in *Pteris vittate,* essentially by dissolving soil arsenic and promoting plant growth [128]. *Enterobacter cloacae* also increased the yield, growth, and photosynthetic pigment content of *Moringa oleifera* Lam. [129]. *Enterobacter hormaechei* KSB-8 significantly increased the fruit maturing, flowering fruit setting, K content, chlorophyll content, and root length of cucumber [130]. Gupta et al. [131] reported that *Enterobacter* sp. strain CPSB49 increased the chromium uptake in the shoots and roots of *Helianthus annuus* L. and increased the plant-growth-promoting ability. Under Cd stress, *Enterobacter* sp. revealed a significant increase in the morpho-biochemical characteristics of seedlings of rice [131].

## 6. *Pseudomonas* spp.

One of the most widespread and diverse bacterial groups in the natural environment is *Pseudomonas* [132]; the genus has significant functions in the soil and biosphere [133], belonging to the phylum *Proteobacteria*, including the major abundant quenching Gram-negative bacteria genus, especially in the rhizospheres of all plant samples [134], being considered as versatile biocontrol agents against bacteria and non-bacterial pathogens [135]. The widespread occurrence of *Pseudomonas* shows its adaptability through physiological, molecular, and environmental diversity. Its family consists of general forms like *Xanthomonas*, *Frateuria*, *Zoogloea*, and *Pseudomonas*. Cyclic lipopeptides from *Pseudomonas mediterranea* have 22 amino acids, which can induce plant dell death immunity and confer resistance to bacterial infection in *Nicotiana benthamiana* [136]. Different strains of *Pseudomonas* show numerous ecological qualified characteristics such as antifungal metabolite production, biofilm formation, synergistic attachment with the plant root system, quorum-sensing mediation, chemotactic mediation, uptake, and the catabolism of different plant secretions. *Pseudomonas fluorescens* UM270 has shown a functional role in the promotion of tomato plant growth, especially under salt stress conditions as it resulted in an increase in dry weight, chlorophyll content, and shoot and root length [137]. The application of *Pseudomonas plecoglossicida* RGK was found to have a capacity to solubilize potassium, zinc, and phosphate, as well as the potential to produce exopolysaccharide, hydrogen cyanide, ammonia, nitrogen fixation, siderophores, and indole acetic acid in turmeric plant [138]. It has also been found to be suitable to improve the growth metrics of plants, such as rhizome biomass, shoot height, the number of leaves, flavonoids, and phenolic content [139]. *Pseudomonas* sp. EB3 can protect banana plants against fusarium and salinity stress, as well as leading to improved growth under non-stressed conditions [140].

In one experiment, *Pseudomonas* sp. RGM 2987 isolated from *Stevia philippiana* roots was introduced as a new plant biostimulant because it showed plant growth promotion characteristics such as IAA, phosphate solubilization, and stress alleviation enzyme production [141]. *Pseudomonas aeruginosa* strain FG106, which was isolated from tomato plants, has shown clear antagonism against *Botrytis cinerea*, *Alternaria alternata*, *Phytophthora colocasiae*, *Phytophthora infestans*, *Xanthomonas euvesicatoria pv. perforans*, *Rhizoctonia solani*, and *Clavibacter michiganensis subsp. michiganensis*, which can also lead to producing siderophores, ammonia, IAA, hydrogen cyanide (HCN), and phosphate solubilization [142]. Chandra et al. [143] reported that *Pseudomonas aeruginosa* from *Valeriana wallichii* shows antagonistic efficacy against *Aspergillus flavus*, *Alternaria alternata*, and *Fusarium oxysporum*. Sahebani and Gholamrezaee [144] also found that *Pseudomonas fluorescens* CHA0 can be used as a potent biocontrol agent to control root-knot nematode, *Meloidogyne javanica*, and the bioinduction of plant defense responses in both cucumber and tomato. Patel et al. [145] reported that *Pseudomonas protegens* Pf-5 and *Pseudomonas* sp. G22 have shown anti-fungal effects against *Magnaporthe oryzae* B157 and *Rhizoctonia solani* in different crops such as wheat, sorghum, and rice. Dignam et al. [146] stated that *Pseudomonas* species diversity explained variation in soil suppressiveness and increased *Pseudomonas* species diversity distinguished as suppressive from conductive soils. Hu et al. [147] also noticed that the multi-strain can increase inoculant abundance in the rhizosphere, as well as plant growth connected with phosphorus solubilization, siderophores, and plant hormones. The effects of different types of *Pseudomonas* on various plants are shown in Table 3.

## 7. *Rhodococcus* spp.

*Rhodococcus* are well known for their significant capacity to degrade various aromatic chemicals, both short- and long-chain, such as heteroaromatic, halogenated, hydroaromatic, and polycyclic aromatic hydrocarbons [222,223,224,225,226]. The genus *Rhodococcus* consists of Gram-positive, aerobic bacteria, and non-sporulating belongs to the phylum *Actinobacteria* [227], which can tolerate extremely toxic components and unfavorable environments because of their cell wall structure and a large array of enzymes that can degrade and toxify harmful components in hostile habitats [228]. They have shown a wide range of metabolic activities [229], and they have been considered because of their biotechnological, agricultural, and ecological importance [230]. Dhaouadi et al. [231] reported that new plant species have been found to be hosts of the plant pathogenic *Rhodococcus fascians* and other newly found members of the genus *Rhodococcus*, and according to their findings, *Rhodococcus* can be found in pistachio and almond trees and root-stocks. *Rhodococcus fascians* can produce a mixture of cytokinins to modify the hormone landscape of its broad range of plant hosts, inducing developmental changes and tissue deformations. Abraham and Silambarasan [232] also found that *Rhodococcus erythropolis* JAS13 could be applied for the integrated bioremediation of pesticides and it plant-growth-promoting capability in agricultural systems. *Rhodococcus erythropolis* MTC 7905 can alleviate Cr^6+^ and promote the plant growth of pea, especially at a low temperature [224]. *Rhodococcus* sp. Fp2 did not stimulate pea growth in Cd-supplemented soil because it had no 1-aminocyclopropane-1-carboxylate deaminase activity in vitro in the presence of Cd [233]. *Rhodococcus* sp. PBTS1 and PBTS2 were able to produce auxins, cytokinins, and plant-growth-stimulating volatiles with notable influences on plant development [234]. In one experiment, it was reported that plant-growth-promoting characteristics were observed after the isolation of *Rhodococcus qingshengii* RL1 from the surface-sterilized leaves of *Eruca sativa* Mill. [235,236].

## 8. Serratia

*Serratia* spp. has various plant growth promotion characteristics, and it stimulates and colonizes the growth of multiple hosts such as non-homologous and homologous forms [237,238,239]. It is a Gram-negative bacterium belonging to *Enterobacteriaceae*, with more than 42 species, and the plant-associated *Serratia* consists of both free-living and endophytic species in the rhizosphere [240]. They can induce root hair development stimulated by their IAA-production and acyl homoserine lactone (AHL) signaling mechanisms [241,242,243]. Lim et al. [244] also found that *Serratia fonticola* DSM 4576^T^ can confer solubilization of inorganic phosphate, hydrogen cyanide production, indole-3-acetic acid production, siderophore production, and assimilation of ammonia via the glutamate synthase (GS/GOGAR) pathway. *Serratia nematodiphila* RGK has been found to have a high capacity to solubilize zinc, phosphate, and potassium, as well as the potency to produce exopolysaccharide synthesis, hydrogen cyanide, ammonia, nitrogen fixation, and the indole acetic acid of the turmeric rhizome of turmeric (*Curcuma longa*). *Serratia* sp. KUJM3 presents various benefits, such as the metalloid bioremediation, plant growth promotion, and As reduction of cowpea [245]. *Serratia marcescens* can stimulate plant growth and increases resistance against *Nilaparvata lugens* in rice, with colonized plants indicating increased seed germination, shoot and root lengths, and shoot and root fresh weights [246]. *Serratia* sp. 5D and RTL100 can be applied as effectual microbial inoculants, especially in nutrient-deficient soils in rainfed areas, where the cultivation of chickpea is common [247]. Devi et al. [248] also concluded that *Serratia marcescens* AL2-16 can increase the growth of latjeera (*Achyranthes aspera* L.), which is one of the most important medicinal plants of the Amaranthaceae family. *Serratia* sp. CP-13 decreases Cd uptake and concomitant lipid peroxidation in maize cultivars, showing its high potential in terms of plant growth augmentation and Cd remediation plans [249]. *Serratia marcescens* AHPC29 can be considered as a new agent for the management of *Bursaphelenchus xylophilus*, which is a destructive and invasive pathogen in forestry [250], with Obi et al. [251] reporting that *Serratia marcescens* 39-H1 was able to increase the hydrolysis of lignocellulosic biomass, being a plant-growth-promoting organism. Bhatta et al. [252] reported that *Serratia marcescens* DB1 is a plant-growth-promoting rhizobacterium with an innate ability to resist heavy metals such as Cr, Ni, and As, which can stimulate the bioavailability of essential elements for plant uptake and keep the balance of Na^+^/K^+^ ions in rice shoots. Ting et al.’s [253] pre-inoculation with the endobacterium *Serratia marcescens* strain UPM39B3 led to the production of host defense enzymes, such as polyphenol oxidase, peroxidase, total soluble phenols, and phenylalanine, in banana plantlets. Zhu et al. [254] found that *Serratia* sp. PW7 can be used to colonize wheat for decreasing pyrene contamination [255], and *Serratia plymuthica* BMA1 can be a potential choice to increase the agronomic effectiveness of *Vicia faba* L. plants toward a clean P-nutrition through the formulation of bio-phosphate fertilizers for plant growth promotion [256]. In another experiment, it was reported that *Serratia marcescens* strain B2 suppressed mycelial growth of the rice health blight pathogen *Rhizoctonia solani* AG-1 IA [257]. Restrepo et al. [257] indicated that *Serratia plymuthica* AED38 extracts showed a promising potential as a bioproduct for the control of avocado root rot caused by *Phytophthora cinnamomi*. *Serratia marcescens* are effectual in increasing the growth and growth characteristics such as leaf Cl^−^, Na^+^ content, and the antioxidant enzyme activities in eggplant under salt stress [258]. Prischmann et al. [259] reported that *Serratia plymuthica* was associated with maize roots and can be considered as a plant-growth-promoting factor through antagonistic action against plant-pathogenic fungi. Youssef et al. [260] also noted that *Serratia proteamaculans* as soil drench effectively increased plant growth and controlled tomato early blight disease. *Serratia marcescens* AS09 was able to reduce disease incidence, promote growth, and increase root length and plant height, and it has shown high potential to be studied as a biocontrol agent against fusarium wilt disease [261].

## 9. Streptomyces

The major genus of *Actinobacteria* is streptomyces, and various strains of streptomyces can promote biocontrol pests and plant growth, weeds, diseases, and phytopathogenic microorganisms by producing phytohormones such as IAA, enzymes, siderophores, antibiotics, volatile organic compounds, and some other secondary metabolites [262,263,264,265]. *Streptomyces* spp. can also alleviate abiotic stresses, such as drought, salinity, and inorganic and organic contaminants in soil, as well as promoting nutrients bioavailability [266,267]. *Streptomyces* are extensively known for the production of an array of components that can promote plant growth directly by phytohormone production such as of cytokinins, indole acetic acid, and gibberellins; through the increased nutrition acquisition of potassium, phosphorus, nitrogen, and essential minerals; or through the suppression of plant diseases [268]. In fact, species of the genus *Streptomyces* are well known as producers of secondary metabolites such as antifungals, antibiotics, anticancer agents, and virulence parameters [269]. Ngalimat et al. [270] reported that *Streptomyces* spp. showed no phytotoxic impact on rice plants, mitigated the negative effects of bacterial panicle blight, increased rice yield attributes, and elicited defense-related gene transcript levels. Zheng et al. [271] illustrated that *Steptomycetes* sp. strain FJAT-31547, presenting broad-spectrum antibacterial and antifungal activity with high biocontrol effectiveness against tomato *Fusarium* wilt and bacterial wilt, was an important growth-promoting factor, as on the basis of GC-MS, n-hexadecanoic acid was recognized as the main constituent of this strain.

The application of *Streptomyces hydrogenans* DH16 significantly increased the shoot length, seed germination, root length, dry and fresh weights, and lateral roots of pea seedlings [272]. The *Streptomyces alfalfae* 11F strain is useful for improving the establishment and biomass yield of switchgrass [273], and *Streptomyces* sp. is appropriate for controlling blueberry canker caused by *Botryosphaeria dothidea* under field conditions [274]. The addition of *Streptomyces parvulus* VRR3 caused a decreased incidence of *Fusarium* wilt disease signs on green gram plant (*Vigna radiata* L.), and it also induced the significant growth of green gram seedlings and enhanced root and shoot length [275]. Park et al. [276] reported that *Streptomyces nigrescens* KA-1 has notable nematicidal activity against *Meloidogyne incognita* with compounds with anti-nematicidal activity that can decrease the number of egg masses and nematodes. *Streptomyces angustmyceticus* strain TH23-7 shows antifungal activity against *Lasiodiplodia theobromae* that indicates its potential to control the spadix rot of flamingo flowers [277]. It has been reported that the usage of *Streptomyces* sp. has a wonderful ability to produce thermostable and acidic phytase, increase plant growth, and promote plant-growth-promoting attributes of tomato plants [278]. The application of *Streptomyces pactum* Act12 in soil increased pepper fruit quality, especially fruit taste, flavor, and color [279]. Abbasi et al. [280] also confirmed that the application of *Streptomyces* can be effectual in commercial bell pepper greenhouses, and that the treated plants showed higher score values of quality characteristics including juiciness, smell, flavor, and appearance. *Streptomyces* strains from the roots of cocoyam can be used for the promotion of root growth and against the root rot disease of *Pythium myriotylum*, which is the main pathogen that limits the productivity and growth of this crop in West Africa [281]. The selected consortium of *Streptomyces* spp. has a higher potential for the biological control of *Fusarium* wilt diseases in chickpea, and its application induces the enhancement of antioxidant enzymes such as catalase, superoxide dismutase, guaiacol peroxidase, ascorbate peroxidase, phenylalanine ammonia-lyase, and glutathione reductase [282]. *Streptomyces* UPMRS4 as a potential bioenhancer and biocontrol agent in rice increased plant growth and upregulated the defense-related genes of rice as well as reducing rice blast disease without meaningful change in yield attributes [283].

*Streptomyces* sp. VITGV100 showed promising antimicrobial activity [284], and *Streptomyces* sp. KRA18-249 indicated strong herbicidal activity against different weeds, being able to be considered as a unique biological factor for weed control [285]. *Streptomyces* sp. CMSTAAHL-3 and its antibiotics can be applied in the treatment of different types of pathogens such as *Pseudomonas aeruginosa*, *Escherichia coli*, *Klebsiella pneumoniae*, *Enterococcus faecalis*, and *Staphylococcus aureus* [286]. The ethyl acetate extract of the *Streptomyces* spp. EGY-S7 strain indicated higher control efficacy against the causing agent of wilt disease of tomato, which is *Fusarium oxysporum*, as well as significantly boosting the biomass of tomato seedlings such leaf number, plant height, and dry and fresh weight. *Streptomyces jietaisiensis* strain A034 can promote plant growth, prevent root knot disease, and keep the soil bacteria community [287], and *Streptomyces* strains can notably increase the growth of wheat in saline conditions [288]. In one trial, *Streptomyces hygroscopicus* OsiSh-2 was able to protect rice seedlings against Fe-deficient stress by a sophisticated interaction with the host, including Fe reduction, solubilization, chelation, and translocation, finally inducing the increased fitness of plants [289]. *Streptomyces corchorusii* strain CASL5 and *Kosakonia radicincitans* strains CABV2 showed high-Al-resistant, improved growth of Al-stressed tomato plants; increased nutrient uptake in plants under Al stress; and declined reduced oxidative stress in seedlings of tomato [290]. The *Streptomyces* spp. strain KPS-E004 and KPS-A032 mixture can promote plant growth, suppress root know infestation, improve the ribotype richness of the bacterial composition in the environment, and the increase yield of chili [291]. Gong et al. [291] demonstrated that *Streptomyces* sp. KLBMP5084 has high potential for promoting tomato seedling growth as the biological fertilizer under salinity stress; they also reported the significant decrease in malondialdehyde, as well as the increasing of proline contents, soluble sugar, and antioxidant enzyme activity in both the stem and leaf of tomato plants. *Streptomyces* sp. strains CBQ-EA2 and CBQ-B-8 can be considered as biological control substitutes against the root rot complex diseases of common bean, as they have shown a high capacity in decreasing disease severity and disease incidence, as well as increasing the yield, germination, and quality of legumes [292]. In another experiment, bacterization with *Streptomyces* spp. reduced the adverse impacts of salinity in corn plants by modulating non-enzymatic and enzymatic antioxidant mechanisms [293]. Nimnoi et al. [294] reported that *Streptomyces galilaeus* strain KPS-C004 can sustain bacterial community, survive long term in the soil, suppress root knot disease, and improve plant growth. *Streptomyces* sp. MBRL 10 and *Streptomyces corchorusii* strain UCR3-16 application can induce a higher germination percentage and significantly increase growth over rice seedlings challenged with pathogens under net house conditions [295].

*Streptomyces roseoflavus* strain NKZ-259 showed high antagonist fungal activity; inhibited the growth of *Botrytis cinerea*; and promoted the growth of pepper and tomato seedlings, especially due to IAA [296]. Toumatia et al. [297] concluded that the highly antagonistic *Streptomyces mutabilis* strain can secrete GA_3_ and IAA to increase wheat seedlings; decrease both decrease severity and disease occurrence of *Fusarium culmorum*; and colonize different niches on the surfaces of the phytosphere, namely, seeds and roots as well as plant endosphere compartments. The inoculation of seeds with *Streptomyces* bacteria stimulated root elongation, increased shoot elongation, and increased cholorophyll levels of sugar beet [298]. *Streptomyces* sp. FXP04 inhibited the mycelial growth of *Phytophthora infestans*, decreased late blight damage on potato plants, and secreted one of the most important polyketide secondary metabolites—Piericidin A [299]. Devi et al. [300] reported that *Streptomyces* sp. SP5 exerted a negative effect on the growth and development of melon fruit fly, which is known as *Zeugodacus cucurbitae*, while Sharma and Manhas [301] proved the positive impacts of *Streptomyces* sp. M4 to increase the resistance power of plants against Alternaria black leaf spot. Basavarajappa et al. [302] observed *Streptomyces* sp. strain SND-2 as a potential source, plant growth promoter, and suppressive agent in mung bean plants, as well as it being a biocontrol agent against anthracnose disease. *Streptomyces pactum* Act12 and *Streptomyces albidoflavus* T4 can increase the grain yield and plant biomass of naked oat, as well as its antioxidant activity [303]. The beneficial characteristics of *Streptomycetes* for plants are related to their capability to produce siderophores and phytohormones auxins, to dissolve unavailable phosphates, and to have ACC deaminase activity [304]. The impacts of different types of *Streptomyces* on experimental plants are shown in Table 4.

## 10. Stenotrophomonas

*Stenotrophomonas maltophilia* linked with plant roots can grow in the availability of different carbon sources such as glucose, chloroform, trichloroethylene, toluene, and benzene, which is also effectual in stimulating plant growth and controlling a wide range of fungal plant pathogens [345]. *Stenotrophomonas maltophilia* is the subclass of γ-β-proteobacteria with a high G + C content, being a Gram-negative bacillus extensively spread in a variety of environmental habitats such as the plant rhizosphere, foods, hospital disinfectant solutions, and soil [346]. It was formerly referred to as *Pseudomonas maltophilia* or *Xanthomonas maltophilia* [347,348]. Its strains can influence and increase plant growth when applied to seedlings, as the strains increase hair development and root growth, and there is always a significant correlation between indole-3-acetic acid and plant growth hormone [349]. *Stenotrophomonas* sp., *Stenotrophomonas chelatiphaga*, *Stenotrophomonas nitritireducens*, and *Stenotrophomonas maltophilia* are among the species that have found to be appropriate in their capability to degrade diverse aromatic components [350]. *Stenotrophomonas maltophilia* is known as the most prevalent organism found in clinical laboratories after *Acinetobacter* spp., *Pseudomonas aeruginosa*, and the *Burkholderia cepacia* complex [351]. Jeong et al. [352] also confirmed that *Stenotrophomonas maltophilia* R13 could be considered as a potential bioinoculant in environments, as well as increasing the nutritional value of feather meal. *Stenotrophomonas* sp. EGS12 has been considered as a wonderful substitute for the bioremediation of repairing a selenium-contaminated environment due to its capability to effectively decrease Se(IV) to form selenium nanospheres [353]. It has been reported that *Stenotrophomonas maltophilia* strain SCS1.1-produces copper nanoparticles, showing its great potency with notable antifungal and antibacterial activity to break down pesticides such as imidacloprid, profenofos, and chlorpyrifos, which are the most well-known organophosphate insecticides against a wide range of pests and insects [354]. *Stenotrophomonas rhizophila* DSM14405^T^ can produce spermidine for both stress protection and growth promotion, and it exerts high Cr(VI) resistance and reductive capability [355]. *Stenotrophomonas maltophilia* strain UN1512 was able to show the causal factor strawberry anthracnose, and its produced volatile compounds can improve tomato seedling growth [356]. Giesler and Yuen [357] reported that *Stenotrophomonas maltophilia* strain C3 prevented the growth of the fungus on leaf blades and decreased the severity of necrosis on seedlings. Li et al. [358] reported that *Stenotrophomonas maltophilia* CGMCC 4254 is an appropriate biocatalyst for the preparation of optically pure L-menthol from diastereomeric mixture. Ercole et al. [359] found that the inoculation of *Stenotrophomonas maltophilia* strains and *Bacillus velezensis* as an important technique to increase salinity tolerance and improve plant growth in maize cultivation.

*Senotrophomonas maltophilia* CR71 and *Pseudomonas stutzeri* E25 can improve the root and shoot length, total fresh weight, and chlorophyll content of tomato plants, as well as being the best choice of biocontrolling *Botrytis cinerea* via the production of potent volatiles like dimethyl disulfide [360]. Feng et al. [361] reported that the application of *Stenotrophomonas pavanii* DJL-M3 as a soil amendment induced relatively little disturbance of fundamental soil functions, such as sulfur and nitrogen cycling, which showed its potency to improve the microbiological environment of rice. *Stenotrophomonas* sp. MNB17 showed high Mn(II) removal capacity because of its function in protein metabolism, electron transport, glyoxylate cycle, and oxidative stress response [362]. Hashidoko et al. [363] introduced *Stenotrophomonas* sp. strain SB-K88 as one of the important antifungal components. Fariman et al. [364] reported that *Stenotrophomonas maltophilia* isolated UPMKH2, which has shown the potential activity to suppress rice blast disease and boost yield. Nigam et al. [365] concluded that *Stenotrophomonas* sp. is a more effectual protectant than salicylic acid for decreasing salinity-caused yield loss because of low-molecular-weight protein profiling, higher APX activity, and higher strength of ionic homeostasis. Alexander et al. [366] reported that *Stenotrophomonas maltophilia* can increase the antioxidant levels, plant growth, scavenging, and stress tolerance of peanut plants under N_2_ deficit conditions. In another experiment, Messiha et al. [367] found it appropriate to control the brown rot of potato caused by *Ralstonia solanacearum*.

Different PGPR like *Ochrobactrum*, *Acinetobacter*, *Arthrobacter*, *Enterobacter*, *Pseudomonas*, *Rhodococcus*, *Serratia*, and *Streptomyces* secrete different phytohormone like gibberellic acid, cytokinin, and auxin. Auxins, majorly IAA also produced by PGPR, have a noticeable impact on overall plant growth promotion, and the main function of IAA is improvement in nutrient uptake, and mineral as well as root system architecture change, which can lead to an overall increase in total root surface and increases in mineral uptake. IAA can increase nodulation, and PGPR significantly improves abiotic stress tolerance. Some of the strains of *Pseudomonas* and *Enterobacter* can increase drought tolerance in plants by reducing abscisic acid concentration and inducing stomatal opening. Different types of PGPR strains can synthesize osmolytes and compatible solutes, which can increase water uptake and maintain osmotic potential. They can also improve the nutrient availability of plants under stress conditions by providing nutrients by different processes such as siderophore production, nitrogen fixation, and potassium and phosphate solubilization. PGPR acidifies the soil microenvironment and significantly influences the soil microenvironment to increase heavy metal tolerance in plants. The mentioned PGPR strain has a notable role in the volatilization and biomethylation of heavy metals, which can increase its availability and mobilization.

In future studies, we suggest a focus on other important PGPB such as *Methylobacterium* sp., *Azospirillum* spp., and *Cynaobacteria*. *Methylobacterium* sp. is a Gram-negative, facultative, straight rod with the ability to use one-carbon compounds as energy and carbon sources, including methylamine and methanol, having a rich heritage of producing plant growth-promoting characteristics such as seed vigor index, seedling length, and the production of growth-promoting phytohormones, being associated with the development and promotion of many crops [368]. Cyanobacteria (blue-green algae) are known to excrete a number of substances that influence plant growth and development; they can produce growth-promoting regulators such as auxin, abscisic acid, cytokinin, gibberellin, vitamins, polypeptides, and amino acids [369]. They can produce a variety of products/compounds and significantly influence plant growth, development, and susceptibility to pathogens [369]. *Azospirillum* is a nitrogen-fixing bacterium found in the rhizosphere of different grass species, as well as among different species of *Azospirillum*, *Azospirillum brasilense*, and *Azospirillum lipoferum*, which are the ones mainly described and studied [370]. As one of the most important PGPR, both *Azospirillum lipoferum* and *Azospirillum brasilense* can increase plant growth by fixing atmospheric nitrogen, as well as through producing plant growth components such as plant hormones, especially auxins [370]. Also, microalgae contain a large group of photosynthetic microorganisms that can produce diverse antioxidant components and colonize harsh environments, and several species of microalgae are known as important biostimulants due to their rich composition in phenolics, polysaccharides, phytohormones, fatty acids, chlorophyll, vitamins, and carotenoids [371]. Microalgal metabolites have been found to impact resistance to plants against abiotic stress, improve soil fertility, improve nutrient uptake from soil, and increase defense response against infection and pathogens [372]. It is also important to consider the important of *Azotobacter* due to its high plant growth promotion activities, nitrogen fixation, heavy metal tolerance, growth hormone production, and pesticide degradation [373,374]. It has been reported that *Azotobacter salinestris* can induce the tolerance of high contents of metal-oxide nanoparticles, and bacterial inoculation increased flower formation, photosynthesis, numbers of fruit, and lycopene content in tomato plants [375], with it having been proposed that the application of *Azotobacter chroococcum* also had significant effects on yam under different levels of nutrient deprivation, whereas it increased the biochemical properties of the final product [376].

*Azotobacter vinelandii* and *Azotobacter chroococcum* rhizobacteria species have significant potential to reduce the negative effects of drought stress by mitigating drought-related oxidative damage in eggplant [377]. The *Azotobacter salinestris* strain could be an alternative tool to increase the production of tomato [378], while *Azotobacter chroococcum* strains can be applied for air-layering for better adaptation in different conditions [379]. *Azotobacter* spp. can also increase the beneficial activities of other biofertilizers if used in consortium, as well as being beneficial in the removal of different stresses [380,381,382]. It is also recommended that more research take place this topic to show the exact mechanisms of *Azotobacter* spp., which can ameliorate plant health and obviate the stressors.

## 11. Conclusions

In the rhizosphere, bacteria produce phytohormones such as cytokinins, ethylene, abscisic acid, gibberellins, and auxin. PGPR can be categorized into free-living rhizobacteria, which live outside plant cells, and symbiotic bacteria, that can be found inside plants and exchange metabolites with them directly. The PGPR exert their impacts through helping to counteract pathogen attack, facilitating food intake, and regulating plant hormone levels. Bacteria of the genus *Arthrobacter* can play an important role in plant growth via direct action mechanisms such as increased iron uptake via iron-chelating siderophores, production of phytohormones, production of volatile components, and solubilization of inorganic phosphates that can influence plant signaling pathways and metabolism. Different biotic stress parameters can influence the growth and reproduction of PGPR in plants. *Pseudomonas* strains are important in the control of different diseases triggered by fungal phytopathogens, such as *Fusarium solani* causing okra root rot, damping off disease caused by *Pythium* spp., foliage blight disease caused by *Phytophthora nicotianae*, and *Rhizoctonia solani* associated with *Rhizoctonia* root-rot. *Pseudomonas stutzeri*, *Pseudomonas fluorescens*, and *Pseudomonas aeruginosa* secrete chitinase and are known as good biocontrol agents, while *Pseudomonas putida* produces different mycolytic enzymes, such as amylase, chitinase, protease, lipase, and cellulase. *Streptomyces* spp. has been found to significantly enhance the intensity of mycorrhizal root colonization in agricultural crops, whereas root colonization by *Streptomyces* in legume crops promotes root nodulation frequency, and *Acinetobacter* can help in plant growth by producing gibberellin, IAA, antibiotics, and siderophore, with an important role in the solubilization of zinc and phosphate. Many strains of *Ochrobactrum* sp. also have a significant role in toxicity mitigation to different crops, especially legume plants. *Rhodococcus* species is a soil-borne organism that is widespread in the environment, especially in soil, having an important function in plant growth as well as a wonderful ability to metabolize harmful environmental pollutants. Different *Serratia* and *Stenotrophomonas* spp. species have plant-growth-promoting abilities that can influence crops by the production of phytohormone indole-3-acetic acid, which has an important role in plant growth promotion. In conclusion, the application of plant-growth-promoting rhizobacteria-based biostimulant bacteria is both an environmentally friendly practice but also a promising methodology that can noticeably improve the use efficiency of natural resources. The main PGPR effects are regulating plant hormone levels, increasing counteraction to pathogen attack, and improving food intake, yet the application of PGPR in the agricultural industry represents a small fraction within the agricultural industry worldwide, which is mainly because of the inconsistent characteristics of the inoculated PGPR that can influence agricultural production. Important parameters that can influence the successful utilization of PGPR are environmental parameters, the interaction capability with indigenous microflora in soil, the compatibility with the crop on which it is inoculated, and their survival in soil. More studies are needed to determine the important effects of PGPR as important parameters to ensure the productivity and the stability of agricultural systems in sustainable agriculture.

## Figures and Tables

**Table 1:** The impacts of different types of *Ochrobactrum* on numerous plants.

Plant	Plant Family	*Ochrobactrum* spp. Type	Key Point	Reference
Apple (*Malus domestica*)	Rosaceae	*Ochrobactrum haematophilum*	It can increase apple growth and degrade phenolic acids, being one of the best treatments for the reduction of apple replant disease.	[44]
Common bean (*Phaseolus vulgaris* L.)	Fabaceae	*Ochrobactrum* sp. Pv2Z2	The inoculation can increase dry and fresh weight, plant height, and nitrogen uptake.	[45,46]
Chili (*Capsicum annuum* L.)	Solanaceae	*Ochrobactrum ciceri*	It is capable of inhibiting the growth of collar rot disease (*Sclerotium rolfsii*) via deteriorating hypha and suppressing the sclerotial formation on broth medium and agar plates.	[47]
Cucumber (*Cucumis sativus* L.)	Cucurbitaceae	*Ochrobactrum* sp. NW-3	It showed a high ability to increase growth and promote plant growth.	[48]
Jerusalem artichoke (*Helianthus tuberosus* L.)	Asteraceae	*Ochrobactrum anthropi* Mn1	It has an important role in root morphological optimization, symbiotic nitrogen fixation, and increased nutrient uptake.	[49]
Lentil (*Lens culinaris*)	Fabaceae	*Ochrobactrum* sp. 42S	It can positively modulate rhizospheric community structure and improve lentil growth.	[50]
Maize (*Zea mays* L.)	Poaceae	*Ochrobactrum* sp. NBRISH6	It can increase overall plant health specially under abiotic stress.	[51]
Rice (*Oryza sativa* L.)	Poaceae	*Ochrobactrum* spp.	It can increase plant growth, as well as improve the nutrient uptake of rice.	[52]
River red gum (*Eucalyptus camaldulensis* Dehnh.)	Myrtaceae	*Ochrobactrum intermedium* BN-3	It has shown high tolerance to Zn, Cd, and Pb, and it can improve the biomass of Pb accumulation.	[53]
Ryegrass (*Lolium perenne* L.)	Poaceae	*Ochrobactrum* sp. PW	It can improve the degradation of pyrene in soil. It can increase the dry weight of ryegrass shoot and root.	[54]
Soybean (*Glycine max* L.)	Fabaceae	*Ochrobactrum* sp. MGJ11	It can secrete IAA and increase tolerance against Cd. It can improve the shoot and root length as well as biomass.	[55]
Sugarcane (*Saccharum officinarum* L.)	Poaceae	*Ochrobactrum intermedium* NG-5	It has shown good biocontrol activity, and it suppresses red rot.	[56]
Tobacco (*Nicotiana tabacum* L.)	Solanaceae	*Ochrobactrum lupini* KUDC1013	It indicated high potential as a biological control against phytopathogens.	[57]
Wheat (*Triticum aestivum* L.)	Poaceae	*Ochrobactrum* spp.	It is the best choice for the solubilization of different P sources.	[58]

**Table 2:** The impacts of *Arthrobacter* spp. on some experimental plants.

Plant	Plant Family	Type of *Arthrobacter* spp.	Key Points	Reference
Cactus pear (*Opuntia ficus-indica* (L.) Mill.)	Cactaceae	*Arthrobacter* sp.	It can improve nutritional and nutraceutical properties of cactus pear, as well as improve growth, yield, and cladode quality.	[83]
Corn (*Zea mays* L.)	Poaceae	*Arthrobacter* sp.	It can support growth in salinity-affected and P-deficient soils.	[84]
Indian pokeweed (*Phytolacca acinosa* Roxb.)	Phytolaccaceae	*Arthrobacter* sp.	It can increase the enhancement of Cd tolerance, as well as improve growth.	[85]
Oregano (*Origanum vulgare* L.)	Lamiaceae	*Arthrobacter* sp. OVS8	It is appropriate in terms of increasing yield and improving the quality of a plant.	[86]
Pea (*Pisum sativum* L.)	Fabaceae	*Arthrobacter protophormiae* (SA3)	It can improve the colonization of beneficial microbes and alleviate salt stress effects and ethylene-induced damage.	[87]
Rapeseed (*Brassica napus* L.)	Brassicaceae	*Arthrobacter globiformis*	It can boost superoxide dismutase enzymatic activities, phenolic compounds, and phenylalanine ammonia-lyase under salt stress.	[88]
Rice (*Oryza sativa* L.)	Poaceae	*Arthrobacter* sp. A2–5	A cold-shock protein (*ArCspA*) from the soil bacterium *Arthrobacter* sp. A2-5 might be involved in the induction of cold-responsive genes and provide cold tolerance.	[89]
		*Arthrobacter* sp.	It can increase rice plant growth.	[90]
Strawberry (*Fragaria* × *ananassa*)	Rosaceae	*Arthrobacter agilis* UMCV2	Its volatile components increase strawberry achene germination and significantly improve strawberry growth in vitro.	[91]
Soybean	Fabaceae	*Arthrobacter* sp. DNS10	It has an important role in terms of the growth, root surface structure, root physiological properties, and leaf nitrogen accumulation of soybean seedlings.	[92]
Sugarcane (*Saccharum officinarum* L.)	Poaceae	*Arthrobacter* sp. strain GZK-1	It can be considered as the best choice for the remediation of s-triazine-polluted agricultural soils.	[93]
Tomato (*Lycopersicon esculentum* L.)	Solanaceae	*Arthrobacter* sp.	It indicated high phosphate solubilizing ability, with great biological activities, and it is an appropriate plant growth promoter.	[94]
		*Arthrobacter* strains TF1 and TF7	It can increase seed germination, vigor index, seedling length, and dry and fresh weight under salt stress.	[95]
Wheat (*Triticum aestivum* L.)	Poaceae	*Arthrobacter* sp. strain C2	It can positively degrade atrazine in mineral salt medium and soil. It can reduce the toxicity of atrazine.	[96]

**Table 3:** The influences of different types of *Pseudomonas* on experimental plants.

Plant	Plant Family	Type of *Pseudomonas* spp.	Key Points	Reference
Amaranthus (*Amaranthus viridis* L.)	Amaranthaceae	*Pseudomonas putida*; *Pseudomonas fluorescence*	It can increase the growth, development, and stress tolerance of plants in humus soil.	[148]
Apple (*Malus domestica*)	Rosaceae	*Pseudomonas fluorescens*	It has the potential to control common postharvest fungal pathogens during storage.	[149]
Arabidopsis (*Arabidopsis thaliana*)	Brassicaceae	*Pseudomonas syringae* effector HopB1	It can increase virulence, but not disease resistance.	[150]
		*Pseudomonas putida* A (ATCC 12633)	It can increase the amounts of proteins in leaf and root biomass.	[151]
		*Pseudomonas syringae*	It has a positive effect on growth and growth development.	[152]
Banana (*Musa acuminata*)	Musaceae	*Pseudomonas aeruginosa* strain Y1 (PaY1)	It is an important activator of banana genes associated with cellular pathways and the hormonal signaling pathway such as detoxification, as well as antioxidant defense, which can increase plant growth and biotic and abiotic tolerance in plants.	[153]
Barley (*Hordeum vulgare*)	Poaceae	*Pseudomonas protegens*	It can significantly improve shoot length, root and shoot fresh weight, and root and shoot dry weight.	[154]
Bean (*Phaseolus vulgaris* L.)	Fabaceae	*Pseudomonas syringae* pv. *phaseolicola*	It is the potential for the biocontrol of halo blight disease.	[155]
Black pepper (*Piper nigrum*)	Piperaceae	*Pseudomonas putida* BP25	It can inhibit a broad range of pathogens such as *Pythium myriotylum*, *Phytophthora capsici*, *Rhizoctonia solani*, *Colletotrichum gloeosporioides*, *Athelia rolfsii*, and *Gibberella moniliformis*, as well as plant parasitic nematode.	[156]
Black cumin (*Nigella sativa* L.)	Ranunculaceae	*Pseudomonas fluorescens* PF1 and PF2	Increases seed production and prevents root rot disease.	[157]
Black mustard seed (*Brassica juncea* (L.) Czern.)	Brassicaceae	*Pseudomonas fluorescens*	Improves plant growth	[158]
Blueberry (*Vaccinium corymbosum* L.)	Ericaceae	*Pseudomonas* spp.	As an important biocontrol agent, it stimulated arthrofactin lipopeptides.	[159]
Cacao tree (*Theobroma cacao* L.)	Malvaceae	*Pseudomonas antagonic*	It is useful in controlling black pod rot caused by Phytophthora palmivora (Butler).	[160]
Canola (*Brassica napus* L.)	Brassicaceae	*Pseudomonas putida*	The inoculation can increase proline content, phenols, and flavonoids; boost glutathione, ascorbate peroxidase, and superoxide dismutase; and improve plant growth in drought stress.	[161]
Castor (*Ricinus communis* L.)	Euphorbiaceae	*Pseudomonas aeruginosa* MAJPJA03	It can increase plant growth and leaf nutrition.	[162]
Chickpea (*Cicer arietinum* L.)	Fabaceae	*Pseudomonas stutzeri*	The inoculation can increase nodule fresh weight, nodulation, and phosphorus uptake.	[163]
		*Pseudomonas citronellolis* KM594397	It increased plant growth and dry biomass under As^5+^ stress.	[164,165]
		*Pseudomonas aeruginosa* strain OSG41	It can increase chemical and biological characteristics in chromium-treated soils.	[166]
Corn (*Zea mays* L.)	Poaceae	*Pseudomonas fluorescens* strain P13	It can increase the growth of corn and degraded phenol in contaminated water and field soils.	[167]
		*Pseudomonas stutzeri* A1501	It can increase the population of indigenous diazotrophs and ammonia oxidizers and functional gene transcripts.	[168]
Cotton (*Gossypium hirsutum* L.)	Malvaceae	*Pseudomonas aeruginosa* Z5	It is the best choice to control the growth of cotton-root-associated fungal pathogens.	[169]
Cucumber (*Cucumis sativus* L.)	Cucurbitaceae	*Pseudomonas* spp.	It can increase cucumber productivity.	[170]
		*Pseudomonas* spp.	Treated seeds showed increased resistance to the damping-off disease caused by *Phytophthora capsici*.	[171]
Eggplant (*Solanum melongena* L.)	Solanaceae	*Pseudomonas* sp. DW1	It can increase SOD activity of the leaves and it can be used as a plant-growth-promoting rhizobacterium.	[172]
Eucalyptus (*Eucalyptus globulus* Labill.)	*Myrtaceae*	*Pseudomonas fluorescens* ECS417	It can control bacterial wilt caused by *Ralstonia solanacearum.*	[173]
Grapevine (*Vitis vinifera*)	Vitaceae	*Pseudomonas protegens* MP12	It has inhibitory effects against phytopathogens such as *Neofusicoccum parvum*, *Penicillium expansum*, *Aspergillus niger*, *Alternaria alternata*, and *Botrytis cinerea*.	[174]
Jerusalem artichoke (*Helianthus tuberosus* L.)	Asteraceae	*Pseudomonas* spp. *Strain* JK2	It has a significant fungicidal impact on *Aspergillus fumigatus*, *Fusarium solani*, and *Aspergillus tamari*.	[175]
Kiwifruit (*Actinidia deliciosa*)	Actinidiaceae	*Pseudomonas syringae pv*. *Actinidiae* (Psa)	It is the causal agent of bacterial canker in kiwifruit.	[176]
Lemon balm (*Melissa officinalis* L.)	Lamiaceae	*Pseudomonas putida*; *Pseudomonas fluorescens*	The seedling inoculation can increase both secondary and primary metabolites.	[177]
Lentil (*Lens culinaris* Medik.)	Fabaceae	*Pseudomonas fluorescens*	It can cause the significant growth of yield and yield components, as well as boost seed nutrient content.	[178]
Lettuce (*Lactuca sativa*)	Asteraceae	*Pseudomonas* spp.	It can increase lettuce growth and improve final yield.	[179]
Madagascar Periwinkle (*Catharanthus roseus* L.)	Apocynaceae	*Pseudomonas fluorescens* RB4	It can result in a higher accumulation of Pb and Cu in shoots, as well as improve the translocation and metal bioconcentration factors.	[180]
Mung bean (*Vigna radiata* L. Wilzeck)	Fabaceae	*Pseudomonas fluorescens*	It can be considered as a biological control of soil-pathogenic nematodes.	[181]
		*Pseudomonas* strain GRP3A	It is an appropriate plant growth promotion under iron-limited conditions.	[182,183]
Olive (*Olea europaea*)	Oleaceae	*Pseudomonas* spp.	Root treatment can help in the biocontrol of *Verticillium* wilt of olives.	[184]
Onion (*Allium cepa*)	Amaryllidaceae	*Pseudomonas alliivorans* sp.	It can improve the plant growth and yield of plants.	[185]
Organo (*Origanum vulgare* L.)	Lamiaceae	*Pseudomonas* spp.	It can improve yield and increase the efficiency of in vitro plant tissue propagation.	[186,187]
Pea (*Pisum sativum* L.)	Fabaceae	*Pseudomonas* spp.	It can control root-knot nematode *Meloidogyne incognita*.	[188]
Peanut (*Arachis hypogaea* L.)	Fabaceae	*Pseudomonas aeruginosa P4*	It can stimulate peanut growth, root system functioning, and defense physiology.	[189]
Pearl millet (*Pennisetum glaucum*)	Poaceae	*Pseudomonas fluorescens*	It can result in the improvement of growth and boost resistance against downy mildew disease caused by the fungus *Sclerospora graminicola*.	[190]
Pepper (*Capsicum annum* L.)	Solanaceae	*Pseudomonas chlororaphis* strain PA23	It can increase enzymes such as polyphenol oxidase, peroxidase, ammonia lyase, and phenol content, as well as decrease the incidence of damping-off	[191]
		*Pseudomonas aeruginosa*	It can increase nutrient uptake and yield parameters, as well as increase the adsorption of phosphorous in the soil.	[192]
		*Pseudomonas putida* BP25	It can increase plant defence responses in pepper roots.	[193]
Pigeon pea (*Cajanus cajan* L.)	Fabaceae	*Pseudomonas* spp.	It can increase plant growth and nutrient uptake.	[194]
Pistachios (*Pistacia vera* L.)	Anacardiaceae	*Pseudomonas* spp. strain VUPf428	It can suppress the root-knot nematodes.	[195]
Rice (*Oryza sativa* L.)	Poaceae	*Pseudomonas fluorescens* strains PF1, TDK1, and PY15	It can control the rice root-knot nematode *Meloidogyne graminicola*.	[196]
		*Pseudomonas fluorescens* PW-5	It can increase root, shoot, and dry weight.	[197]
*Sedum alfredii* Hance	Crassulaceae	*Pseudomonas fluorescens*	It can increase the reversed and long-distance transport of Cd and sucrose in the plant by examining the phloem and xylem sap, as well as by quantifying the contents of sucrose and Cd in root and shoot.	[198]
Sesame (*Sesamum indicum* L.)	Pedaliaceae	*Pseudomonas fluorescens*	It can increase plant growth and enhance crop production.	[199]
Sorghum (*Sorghum bicolor L*.)	Poaceae	*Pseudomonas* sp. *P17*	It is considered as a potential plant growth promoter.	[200]
		*Pseudomonas fluorescens*	It has a meaningful influence on the root and shoot length of sorghum.	[201]
Soybean (*Glycine max*)	Fabaceae	*Pseudomonas putida* KT2440	It can significantly increase seed germination, root and shoot length, and dry and fresh weight of plants.	[202]
		*Pseudomonas parafulva* JBCS1880	It can be considered as a specific biological control agent.	[203]
		*Pseudomonas putida* H-2-3	It can induce tolerance against abiotic stress and also cause an increase in antioxidants.	[204]
Sugarcane (*Saccharum officinarum* L.)	Poaceae	*Pseudomonas* spp.	It can be used for the management of red rot disease in sugarcane-growing districts.	[205]
Sunflower (*Helianthus annuus* L.)	Asteraceae	*Pseudomonas monteilii*; *Pseudomonas aeruginosa*	It can significantly suppress the root rotting fungi of sunflower and improve fresh shoot weight and plant height.	[206,207]
		*Pseudomonas lurida* strain EOO26	It can increase the length and dry weight of shoot and root of sunflower and increased Cu uptake.	[208]
		*Pseudomonas* sp. AF-54	It can increase sunflower crop yield.	[209]
Thale cress (*Arabidopsis thaliana*)	Brassicaceae	*Pseudomonas aeruginosa* PA01	It can improve the ability of the plant in terms of different abiotic and biotic parameters that may cause stress.	[210]
Tobacco (*Nicotiana tabacum*)	Solanaceae	*Pseudomonas* sp. TK35-L	It can promote tobacco root development by upregulating the transcript levels of the *HRGPnt3* gene, which can promote tobacco seedling growth.	[211]
		*Pseudomonas* spp.	It can increase the Ca-phytate bioavailability to tobacco up to 10-fold.	[212]
Tomato (*Solanum lycopersicum* L.)	Solanaceae	*Pseudomonas putida* SAESo11	Its inoculation can adapt plants to drought stress and keep the redox state of plants at exposure to drought stress.	[213]
		*Pseudomonas* spp.	Its application can help provide enough Zn bioavailability that can be induced to increase plant growth in a sustainable manner.	[214,215]
		*Pseudomonas chlororaphis*; *Pseudomonas fluorescens*	It can be effective against *Salmonella* strains as a post-harvest application.	[216]
		*Pseudomonas fluorescens* G20-18	It can increase drought stress and contribute to increasing the robustness of the practical utilization of it to promote crop resilience.	[217]
		*Pseudomonas oryzihabitans* PGP01	It has positive effects on the roots of plants.	[218]
		*Pseudomonas chlororaphis* subsp. *aureofaciens* strain M71	Its inoculation can increase the ABA level in leaves of water-stressed tomatoes.	[219]
		*Pseudomonas Syringae*	It can increase the plant defense system.	[220]
Turmeric (*Curcuma longa*)	Zingiberaceae	*Pseudomonas fluorescens* FP7 and TPF54	It can increase the defense molecules, yield, and plant growth in turmeric plants and finally reduce the incidence of rhizome rot diseases caused by *Pythium aphanidermatum*.	[221]
Wheat (*Triticum aestivum* L.)	Poaceae	*Pseudomonas* spp.	It can lead to increased grain yield.	[222]

**Table 4:** The effects of different types of Streptomyces on experimental plants.

Plant	Plant Family	Type of *Streptomyces*	Key Points	Reference
Alfalfa (*Medicago sativa* L.)	Fabaceae	*Streptomyces* spp.	The strains can decrease defoliation caused by fungal plant pathogen (*Phoma medicaginis* var. *medicaginis*).	[305]
Banana (*Musa acuminata*)	Musaceae	*Streptomyces* sp. H4	It can improve the growth of banana seedlings, and it is an important microbial resource for the biocontrol of banana *Fusarium* wilt caused by *Fusarium oxyspoum* f. sp. *cubense* (Foc).	[306]
Chickpea (*Cicer arietinum* L.)	Fabaceae	*Streptomyces* spp.	It can trigger systemic resistance in chickpea under *Sclerotium rolfsii* stress though increasing different enzymes.	[307]
		*Streptomyces* spp.	It has the potential for the biological control of *Botrytis cinerea* in chickpea.	[308]
		*Streptomyces* spp.	It is appropriate to control *Fusarium* wilt disease and increase plant growth.	[309]
Common comfrey (*Symphytum officinale*)	Boraginaceae	*Streptomyces pactum*	Its application can lead to decreased Cd and Zn concentration in shoots.	[310]
Cucumber (*Cucumis sativus* L.)	Cucurbitaceae	*Streptomyces roche* D74; *Streptomyces pactum* Act12	Its applications can be applied to reassemble and optimize the rhizosphere microbiome of plants, which can induce to plant survival.	[311]
		*Streptomyces goshikiensis* YCXU	It showed antifungal activity against fungal pathogens, and it reduced the incidence of *Fusarium* wilt.	[312]
		*Streptomyces* sp. C-11 and C-26	It showed a high ability to produce enzymes, such as lipase, protease, and amylase.	[313]
Ginseng (*Panax ginseng*)	Araliaceae	*Streptomyces werraensis* F3	It has a great potential as a biological control agent to effectively manage ginseng rust rot and root rot diseases.	[314]
Grapes (*Vitis vinifera* L.)	Vitaceae	*Streptomyces plumbeus* strain CA5	It inhibited gray mold development on grapes.	[315]
		*Streptomyces alni*	It is a biocontrol agent for controlling the root-rot of grapevine and other soil-borne plant pathogens.	[316]
Maize (*Zea mays* L.)	Poaceae	*Streptomyces* sp. M7	It can positively influence the vigor index and germination of maize plants.	[317]
Melon (*Cucumis melo* L.)	Cucurbitaceae	*Streptomyces* spp.	It can be used as a biocontrol of gummy stem blight (*Didymella bryoniae*) and improve the growth of a plant.	[318]
Mung bean (*Vigna radiata* L.)	Fabaceae	*Streptomyces* sp. GMKU 336	It can significantly increase biomass and plant elongation, leaf color, leaf area, chlorophyll content, and adventitious roots, as well as decrease the ethylene level.	[319]
		*Streptomyces* spp.	It can suppress the root rot of mung bean	[320]
Oil palm (*Elaeis guineensis*)	Arecaceae	*Streptomyces* sp. GanoSA1	It can induce to a higher vegetative index, lower diseases incidence, reduced mortality, and mitigated severity of foliar symptoms, as well as act as a biological control agent.	[321,322]
		*Streptomyces palmae*	A potential biocontrol agent for basal stem rot disease caused by *Ganoderma boninense*.	[323]
Oilseed rape (*Brassica napus* L.)	Brassicaceae	*Streptomyces* spp.	It is a promising agent to improve the growth of oilseed rape.	[324]
		*Streptomyces platensis* 3–10	It is a promising biocontrol agent against *Plasmodiophora brassicae*, the causal agent for clubroot of oilseed rape.	[325]
Pea (*Pisum sativum* L.)	Fabaceae	*Streptomyces* spp.	It can be used for the management of foot rotting and blight caused by *Mycosphaerella pinodes*.	[326]
Peanut (*Arachis hypogaea* L.)	Fabaceae	*Streptomyces* sp. RP1A-12	It has shown to have high effects in decreasing the incidence and severity of stem rot caused by *Sclerotium rolfsii*.	[327]
Pepper (*Capsicum* sp.)	Piperaceae	*Streptomyces* spp.	It can fight against the development of phytophthora blight on Chile pepper.	[328,329]
Pomegranate (*Punica granatum* L.)	Punicaceae	*Streptomyces* spp.	It can increase final yield and chemical components.	[330]
Rice (*Oryza sativa* L.)	Poaceae	*Streptomyces* spp. AB131-1	It induced to the highest plant height, increased the number of tillers, and can be used as a biocontrol agent against rice bacterial leaf blight pathogen (*Xanthomonas oryzae* pv. *oryzae*).	[331]
		*Streptomyces* spp.	The Iranian strain of *Streptomyces* has antifungal properties for the control of the rice sheath blight disease, because of *Rhizoctonia solani* (AG1-IA).	[332]
		*Streptomyces albidoflavus* OsiLf-2	It can increase stress responses in the rice host at the biochemical and physiological levels, as well as cause significant tolerance to salinity.	[333]
		*Streptomyces hygroscopicus* OsiSh-2	It is used to cope with *Magnaporthe oryzae* (Mo)-toxins in rice.	[334]
		*Streptomyces hygroscopicus* OsiSh-2	It is a potential biocontrol agent against the rice blast pathogen.	[335]
Strawberry (*Fragaria* × *ananassa*)	Rosaceae	*Streptomyces* spp. MBFA-172	It can effectively suppress strawberry anthracnose caused by *Glomerella cingulata.*	[336]
		*Streptomyces* sp. H4	It has shown anti-fungal activity with high effectiveness in controlling the anthracnose of strawberry fruit.	[337]
		*Streptomyces hygroscopicus* B04	It is a potential biocontrol agent for controlling strawberry root rot.	[338]
Sugar beet (*Beta vulgaris* L.)	Amaranthaceae	*Streptomyces* spp.	It has the potential for application as a biocontrol agent against fungal diseases, particularly in saline soils.	[339]
Tomato (*Solanum lycopersicum* L.)	Solanaceae	*Streptomyces pactum*	It can decrease the emergence of *Phelipanche aegyptiaca* and promote host defense mechanisms.	[340]
		*Streptomyces* spp.	It can decrease root knot nematode disease in tomato by activating defensive mechanisms and systemic resistance against nematode infection.	[341]
Wheat (*Triticum aestivum* L.)	Poaceae	*Streptomyces* spp.	It can improve crop production and ensure plant health protection.	[342]
		*Streptomyces pactum* (Act12)	Its addition increased Zn, Cu, and Cd in the roots and shoots, and P in the roots. It can also decrease lipid peroxidation and antioxidant activities in wheat.	[343]
		*Streptomyces badius*	It is an important source for the reduction of chemical fertilizers.	[344]
